# Genomic and Phenotypic Classification of *Staphylococcus* Species Isolated From Delhi Air

**DOI:** 10.1111/1758-2229.70411

**Published:** 2026-09-15

**Authors:** Himanshu Sen, Joyasree Das, Suman Paul, Manpreet Kaur, Amareshwar Vodapalli, Surajit Bhattacharjee, Manoj Raje, Srinivasan Krishnamurthi, Saumya Raychaudhuri

**Affiliations:** ^1^ CSIR‐Institute of Microbial Technology Chandigarh India; ^2^ Academy of Scientific and Innovative Research (AcSIR) Ghaziabad India; ^3^ Department of Molecular Biology & Bioinformatics Tripura University (A Central University) Tripura India; ^4^ Department of Biophysics Panjab University Chandigarh India

## Abstract

To understand the prevalence of opportunistic bacterial pathogens in the bioaerosols, outdoor air‐sampling was performed during the peak season of air pollution prior to the onslaught of the COVID‐19 pandemic in the national capital of India, Delhi, during the month of November 2019. Employing a sophisticated plate exposure technique using the Merck Mas Eco‐100 instrument, culturable bacterial samples were collected from six locations in Delhi. Among the identified bacterial samples, multiple species of the opportunistic pathogen *Staphylococcus*, namely, *S. gallinarum* , *S. haemolyticus* , and *S. arlettae* were present. To further characterize these isolates, whole genome sequencing coupled with phenotypic microarray, antibiotic resistance profiling, and biofilm formation in the presence of a known air pollutant, black carbon, was performed. The comparative genomics analysis highlighted the presence of the urease gene cluster (*ureABCDEFG*) in *S. arlettae* Naraina and *S. haemolyticus* Yojna Vihar 1 that may provide a fitness advantage to these strains, as urease activity in *S. haemolyticus* and *S. arlettae* is usually reported as negative.

## Graphical abstract

Multiple *Staphylococcus* spp. were isolated from the outdoor polluted air of the Indian Capital, New Delhi. The bacterial samples were collected from 6 different locations, and the whole genome of four *Staphylococcus* isolates was sequenced in their entirety. This study presents the comparative genomic analysis of these four isolates.

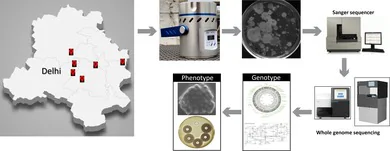

## 1 Introduction

The field of Aeromicrobiology, a branch of environmental microbiology, is gaining tremendous momentum, especially after the COVID‐19 global pandemic, for better understanding of the impact of microbes present in the air on human health (Atta 2020). Notwithstanding the substantial advancements in addressing air pollution stemming from particulate matter (PM) and its ramifications for global health, the influence of airborne microbiological entities, collectively known as the “atmospheric microbiome,” and related molecules such as bacterial endotoxin on the global health index is an area whose deeper exploration is still in its nascent stages (Soukup and Becker 2001; Salim et al. 2014; Morales‐Bárcenas et al. 2015; Chirino et al. 2017).

PM is the main constituent of air pollution (Kelly and Fussell 2011, 2012). It is made up of solid particles and liquid droplets in the air. Based on the size, PM is categorized into three groups, for example, coarse particles (PM10), fine particles (PM2.5), and ultrafine particles (PM0.1) (Kelly and Fussell 2012). It has now been well established and recognized how PM, as per its size, adversely impacts the health index, especially respiratory diseases from mild to severe chronic obstructive pulmonary disease (COPD) (Soukup and Becker 2001; Morales‐Bárcenas et al. 2015; Chirino et al. 2017). Not only respiratory, but also systemic diseases, including inflammatory as well as gut‐related diseases, are also caused by air pollution through the modulation of gut microbiome (Lundborg et al. 2007; Heal et al. 2012; Host 2013; Salim et al. 2014; Rylance et al. 2015). Chemical analysis of PM reveals black carbon (BC) as a major component (Dominici et al. 2014; Pan et al. 2022). Interestingly, Morrissey and colleagues have clearly demonstrated how BC alters biofilm development of two notorious pathogenic bacteria *Staphylococcus aureus* and *Streptococcus pneumoniae* , thereby increasing their tolerance to multiple antibiotics. In a mouse model, BC also increases colonization of these pathogens (Hussey et al. 2017). Taken together, PM severely impacts the outcome of infectious and lifestyle diseases.

Other than PM, bioaerosols also contribute significantly to air pollution. Bioaerosols are biogenic particles, such as bacteria, fungal spores, viruses, pollen grains, endotoxins, mycotoxins, and other biological materials, in the air (Douwes et al. 2003; Mandal and Brandl 2011). In recent years, exposure to bioaerosols in occupational settings has gained significant interest because of adverse health outcomes (Kim et al. 2018). For example, workers from the waste recycling industry, food processing industry, and detergent industry are often exposed to high concentrations of microorganisms and microbial byproducts in the form of bioaerosols. The continuous exposure causes severe inflammatory and respiratory distress among workers (Van Tongeren et al. 1997; Thorn and Rylander 1998; Douwes et al. 2000; Schweigert et al. 2000; Wouters et al. 2002). While we are gaining an understanding of the effects of bioaerosols in occupational and residential environments, information on these particles in an urban environment, especially in India, is very limited.

We embarked on the present project to gain insights into the status of biogenic aerosols in the air of the Delhi megacity. Delhi was chosen as it is the capital of India, and the air quality of Delhi poses a huge public health threat (Chen 2025). The air samples were collected from six congested places in Delhi. Microbial analysis of each sample was carried out. Representatives of 11 different genera were found in over a hundred isolates. Out of those bacterial isolates, we performed genomic characterization of *Staphylococcus* species. We also conducted an antimicrobial resistance profile as well as phenotypic diversity analysis of these isolates. Collectively, our data illustrated the presence of multiple virulence and antibiotic resistance genes in all four sequenced *Staphylococcus* spp. genomes. Moreover, their biofilm‐forming potential was also checked in the presence of BC particles (one of the major air pollutants in outdoor air).

## 2 Materials and Methods

### 2.1 Bacterial Sample Collection and Pure Culture Preservation

**FIGURE 1:**
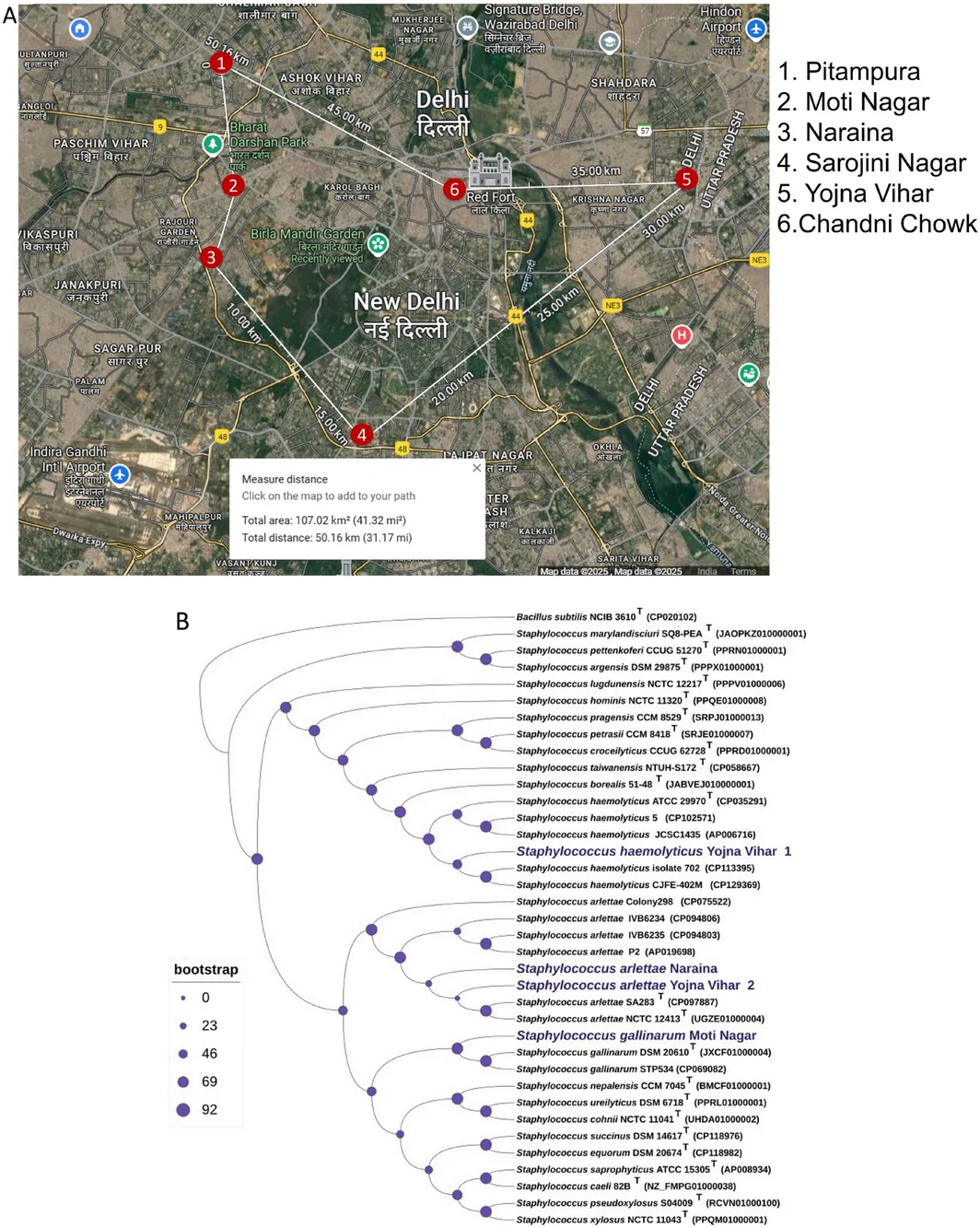
Location and species mapping in their respective map/tree. (A) Mapping the various sampling sites in Delhi using Google Maps. (B) Phylogenetic tree based on 92 single copy core genes. The tree was constructed using UBCG pipeline and visualized in iTOL (https://itol.embl.de/).

Sampling to isolate bacterial and fungal cultures from the outdoor air of Delhi, India was conducted post‐Diwali autumn season of 2019. Sampling was conducted at six sites of the megacity, in triplicate. Sampling sites consisted of different locations, including the densely populated Chandni Chowk area, the heavy traffic region of Pitampura, roof‐top of a public school in Sarojini Nagar, the industrial area near the railroads in Naraina, and residential buildings in Moti Nagar and Yojna Vihar (Figure 1A). This approach was employed to increase the culturable biodiversity in Delhi. The air sampling was carried out using the MAS‐100 Eco air sampler (Merck, Darmstadt, Germany) on tryptic soy agar (TSA) plates (supplemented with amphotericin B at 2 μg/mL). Media components were purchased from HiMedia (Mumbai, India). At each sampling site, the device was positioned at 1.5 m above the ground using a tripod, and the airflow was set to 100 L per minute. Each plate was inoculated with 300 L of air and incubated at 37°C for 48 h.

Morphologically distinct colonies were then streaked and passaged to obtain pure cultures and stored in glycerol at −80°C. Pure cultures were grown in Lysogeny broth (LB) for DNA isolation and Mueller‐Hinton (MH) broth for antibiotic susceptibility testing.

### 2.2 DNA Isolation, 16S rDNA Sequencing and Sequence Alignment

Genomic DNA of the pure cultures was isolated using the Zymo Quick‐DNA Fungal/Bacterial Miniprep Kit (Cat#D6005) and the company protocol. 16S rDNA of all isolates was then amplified using the 27F (5′‐AGAGTTTGATCCTGGCTCAG‐3′) and 1492R (5′‐CTACGGCTACCTTGTTACGA‐3′) universal primers with Promega's GoTaq Mastermix (Cat#M7122). The 16S rDNA amplicons were then sequenced using the automated DNA sequencer, Applied Biosystems 3130/3130*xl*.

DNA sequences obtained were then aligned with the available sequences on the Genbank using BLAST (Basic Local Alignment Search Tool) (https://blast.ncbi.nlm.nih.gov/Blast.cgi). Genus and species identification was confirmed using this analysis.

### 2.3 Genome Sequencing, Assembly and Annotation

The complete genome sequencing of four air inhabitant *Staphylococcus* spp. was conducted using a hybrid strategy with a combination of PacBio long reads and Illumina short reads. Briefly, for PacBio HiFi reads, after purification, SMRT library was prepared, followed by size selection analyses using Agilent FEMTO pulse analyser (Agilent Technologies, California, USA). The library was constructed using the SMRTbell Express template Preparation Kit 3.0 (Pacific Biosciences, California, USA) as per the manufacturers' protocol. The prepared library was purified using AMPure PB beads (Pacific Biosciences, California, USA). AMPure PB bead size selection was carried out to remove fragments shorter than 5 kb. Selected SMRT libraries were purified, then subjected to primer annealing, polymerase binding using Sequel II binding kit 3.2 to prepare bound complex. About 90 pM of the libraries were loaded on SMRTcell containing 8 M ZMW and sequenced by PacBio Sequel II system in CCS/HiFi mode. While for short reads, the libraries were prepared using KAPA HyperPrep Kit for Illumina sequencing, (Cat# 7962363001). Prepared libraries were quantified using Qubit HS Assay (Invitrogen, Cat# Q32854). The pooled libraries were then loaded onto Illumina HiSeq instrument to generate 3.5 Gb, 150 bp paired‐end reads/sample. The long reads and short reads were pre‐processed using filtlong (v0.2.0) and fastp (v0.21.0), respectively, with default parameters (Chen et al. 2018; Wick and Menzel 2019). The assembly of the four strains was performed using Flye v2.9.3 (Kolmogorov et al. 2019) with default parameters. Subsequent polishing of assembled genomes was performed using polypolish followed by pilon (Walker et al. 2014; Wick and Holt 2022). Genome completeness and contamination were checked using CheckM2 v1.2.0 (https://github.com/chklovski/CheckM2, Chklovski et al. 2023). Gene prediction and functional annotation were conducted using Backta with default parameters (Schwengers et al. 2021). The genome‐genome alignment was performed using Mauve (v2.3.1), and a circular plot of plasmids was drawn using Proksee (Grant et al. 2023). The FNA (nucleotide sequence) files of all the *Staphylococcus* spp. were retrieved from GenBank, and phylogenetic analysis was carried out with genome sequences of strains Moti Nagar, Yojna Vihar 1, Naraina, and Yojna Vihar 2 based on 92 core genes using UBCG pipeline (Na et al. 2018).

To estimate overall genome‐related indices, *in silico* DNA–DNA hybridization (dDDH) was calculated using the Genome‐to‐Genome Distance Calculator (GGDC 3.0) by the Type (Strain) Genome Server (TYGS), accessible at [https://ggdc.dsmz.de/ggdc.php#] (Meier‐Kolthoff et al. 2013, 2022). Additionally, average nucleotide identity (ANI) was determined using the ANI Calculator (Lee et al. 2016). Secondary metabolite‐producing gene clusters (BGCs) were annotated using the antiSMASH tool (v7.0) (Blin et al. 2023). The antibiotic resistance genes were analysed with the Resistance gene identifier against the Comprehensive Antibiotic Resistance Database (CARD) (https://card.mcmaster.ca/analyze/rgi). Further genomic islands were predicted with IslandViewer 4, insertion sequences were predicted using IS finder (https://isfinder.biotoul.fr/), phage annotation was conducted using PHASTER (https://phaster.ca/), and virulence genes were predicted using VFanalyzer (Siguier et al. 2006; Arndt et al. 2016; Bertelli et al. 2017; Liu et al. 2019).

### 2.4 Antimicrobial Susceptibility Testing

All *Staphylococcus* isolates were tested for antibiotic susceptibility using the standard Kirby‐Bauer disk diffusion method against 7 classes of antimicrobials at their breakpoint concentration in accordance with the Clinical and Laboratory Standards (Weinstein and Lewis 2nd 2020). The 7 classes of antibiotics used were β‐lactams (ampicillin, amoxiclav, ceftriaxone, cefepime, meropenem, methicillin), aminoglycosides (kanamycin and gentamicin), tetracycline, sulfonamides (sulphafurazole, and co‐trimoxazole), fluoroquinolone (ciprofloxacin), chloramphenicol, and glycopeptide (vancomycin). The antibiotic disks were procured from HiMedia (Mumbai, India).

### 2.5 Determination of Minimum Inhibitory Concentration (MIC)

The MIC of vancomycin against four *Staphylococcus* spp. isolates was determined by broth microdilution (BMD) in accordance with the Clinical and Laboratory Standards Institute (CLSI) guidelines (CLSI 2024; M100, 34th ed.). Briefly, two‐fold serial dilutions of vancomycin were prepared in cation‐adjusted Mueller‐Hinton broth (CAMHB) to yield a concentration range of 0.125–64 μg/mL in sterile, flat‐bottomed 96‐well microplates. Bacterial inoculums were prepared from overnight cultures adjusted to a 0.5 McFarland turbidity standard and subsequently diluted in CAMHB to achieve a final concentration of 5 × 10^5^ CFU/mL per well. Plates were incubated aerobically at 37°C for 24 h. The MIC was recorded as the lowest concentration of vancomycin at which no visible turbidity was observed. Results were interpreted using CLSI M100 breakpoints: susceptible ≤ 4 μg/mL; intermediate 8–16 μg/mL; resistant ≥ 32 μg/mL. All assays were performed in biological triplicate with two independent experimental repeats.

### 2.6 Biofilm Development and Exopolysaccharide Production by the Staphylococcus Isolates

Overnight‐grown cultures of all four *Staphylococcus* spp. ( *S. arlettae* Naraina, *S. arlettae* Yojna Vihar 2, *S. gallinarum* Moti Nagar and *S. haemolyticus* Yojna Vihar 1) were diluted in brain heart infusion broth (BHI) to an OD600 nm of 0.5 and were inoculated in BHI with sterile glass slides placed in 12‐well plates. Cultures were grown with or without BC (100 μg/mL) for 48 h at 37°C for biofilm formation. Post incubation, planktonic cells and media were removed, and biofilms were washed with PBS followed by fixation with 2.5% glutaraldehyde. Fixed cells were then rinsed with 0.1 M Sörensens buffer and dehydrated using an ethanol gradient. For SEM, the biofilms were gold‐coated and viewed on Carl Zeiss Sigma 500 FEG‐SEM.

The production of exopolysaccharide (EPS) by all four staphylococci was measured. The EPS was extracted from four isolated *Staphylococcus* spp. following published protocols (Goswami et al. 2025; Paul et al. 2026). Briefly, isolates were grown in LB medium on glass coverslips in petri dishes with and without BC 50 μg/mL for 48 h at 37°C under static conditions. Biofilms were washed twice with 1X PBS, scraped, and centrifuged (3500 × g, 20 min, 4°C). The supernatant was collected, and the pellet was treated with 10 mM EDTA, vortexed (15 min), and recentrifuged. Both supernatants were pooled, mixed with 2.2 volumes of chilled absolute ethanol, and incubated at −20°C for 1 h, followed by centrifugation. The EPS pellet was dissolved in sterile distilled water, and carbohydrate content was quantified by the phenol sulphuric acid method (OD 490 nm) (DuBois et al. 1956).

### 2.7 Biolog Plates and Analysis

The biochemical characteristics of the four *Staphylococcus* strains, namely *S. arlettae* Naraina, *S. arlettae* Yojna Vihar 2, *S. gallinarum* Moti Nagar, and *S. haemolyticus* Yojna Vihar 1, were carried out using the Biolog GEN III MicroPlate identification system (Biolog, USA) following the manufacturer's instructions. Briefly, inoculation fluid B (Biolog, USA) was used to prepare a suspension of each strain, and the turbidity was adjusted to 90%–98% transmittance using a turbidometer (Biolog, USA). Subsequently, 100 μL of cell suspension was inoculated into each well of the GEN III microplate and incubated at 37°C for 24 h. The results were obtained using Biolog MicroStationTM (Biolog, USA).

## 3 Results

### 3.1 Sample Collection, Isolation, and 16S rRNA‐Based Identification of *Staphylococcus* spp

The air samples were collected from six different locations in the Delhi megacity to test the culturable microbial diversity of the Delhi outdoor air. The locations selected were Naraina (28.634026593199494, 77.13510979747053), Yojna Vihar (28.664204684708672, 77.31735909979705), Pitampura (28.69448263635697, 77.15006383241901), Chandni Chowk (28.657098165172947, 77.22760945130197), Sarojini Nagar (28.574910347940218, 77.19235062815355), and Moti Nagar (28.658166847485457, 77.14294405770747) (Figure 1A). The samples were subjected to microbial isolation and identification by employing classical and molecular approaches. Initial screening with 16S rDNA sequencing followed by BLAST analysis and identification using Biolog Gen III Microplate highlighted the presence of pathogenic *Staphylococcus* species in our samples isolated from three of the six sampling sites, namely Moti Nagar, Naraina, and Yojna Vihar (Figure S1). The isolated *Staphylococcus* spp. were identified as *S. gallinarum* from Moti Nagar, *S. haemolyticus* and *S. arlettae* from Yojna Vihar, and *S. arlettae* from Naraina. Understanding the rising concern of these *Staphylococcus* spp. hovering in Delhi air further propelled us to investigate at the genomic level.

The isolates were named as *S. arlettae* Naraina, *S. arlettae* Yojna Vihar 2, *S. haemolyticus* Yojna Vihar 1, and *S. gallinarum* Moti Nagar. Keeping view of their pathogenic potential (Kolawole and Shittu 1997; Barros et al. 2012; Dinakaran et al. 2012; Lavecchia et al. 2019; Shi et al. 2019; Eltwisy et al. 2020; Han et al. 2023), these isolates were further investigated at the genome level in order to gain additional insight into their virulence potential. Thus, the whole genome sequencing of these four strains was conducted.

### 3.2 Genomic Properties of Air‐Borne Staphylococcus spp

The complete genome sequencing of the four air‐inhabitant *Staphylococcus* spp. was conducted using a hybrid strategy with a combination of PacBio long reads and Illumina short reads. Each of the four genomes was assembled into single contigs ranging from 2.5 to 2.9 Mb with a GC content of 33.0%–33.7%. The assembly coverage ranged from 70 to 79×. Further, the quality of the assembled genomes was evaluated using CheckM2, and estimated completeness was > 99%, and contamination levels were < 0.5%. Subsequently, Bakta‐based annotation identified 2679, 2419, 2409 and 2452 coding genes (CDS) in *S. gallinarum* Moti Nagar (CP182780), *S. arlettae* Yojna Vihar 2 (CP182778), *S. haemolyticus* Yojna Vihar 1 (CP182779) and *S. arlettae* Naraina (CP182784) genomes respectively, among which 52, 28, 47 and 32 were hypothetical proteins (Table 1). Additionally, two plasmid DNA sequences in the *S. arlettae* strain Yojna Vihar 2 [P1_YV2 (CP182776), P2_YV2 (CP182777)] and three in *S. gallinarum* Moti Nagar [namely P1_MB (CP182781), P2_MB (CP182782), P3_MB (CP182783)] were detected. The detailed assembly and annotation features were presented in Table 1.

**TABLE 1:** Genome assembly and annotation summary of four *Staphylococcus* spp.

Strains	Contigs	N50	L50	Genome size	GC%	tRNA	rRNA	CDS	Hypothetical genes	Pseudo genes	Coding density
Chromosome											
*S. gallinarum* Moti Nagar	1	2,865,853	1	2,865,853	33.4	60	22	2679	52	13	85.3
*S. arlettae* Yojna Vihar 2	1	2,572,429	1	2,572,429	33.7	60	22	2419	28	5	85.9
*S. haemolyticus* Yojna Vihar 1	1	2,514,542	1	2,514,542	33.0	62	19	2409	47	16	87.8
*S. arlettae* Naraina	1	2,610,696	1	2,610,696	33.6	60	22	2452	32	6	85.9
Plasmid											
P1_MB	1	29,342	1	29,342	29.0	—	—	14	2	0	86.5
P2_MB	1	20,258	1	20,258	27.7	—	—	20	2	0	80.8
P3_MB	1	27,379	1	27,379	29.2	—	—	31	1	0	81.1
P1_YV2	1	27,660	1	27,660	30.5	—	—	32	1	0	76.8
P2_YV2	1	28,757	1	28,757	28.2	—	—	29	9	0	83.8

The 92‐core gene‐based phylogenetic tree of these four strains with the closest *Staphylococcus* spp. revealed that strain Moti Nagar clusters with *S. gallinarum* , strain Yojna Vihar 1 with *S. haemolyticus* , and the two strains Naraina and Yojna Vihar 2 were clustered with *S. arlettae* (Figure 1B). The overall genome related indices of strains Moti Nagar, Yojna Vihar 1, Naraina, and Yojna Vihar 2 show dDDH identity values of 93.9%, 77.2%, 92.2%, and 93.4% and ANI values of 99.3%, 97.3%, 99.1%, and 99.3% respectively (Table 2) with the reference genomes of type strain *S. gallinarum* DSM 20610^T^, *S. haemolyticus* ATCC 29970^T^, *S. arlettae* NCTC 12413^T^ respectively based on the fact that higher than threshold GGDC (≥ 70%) and ANI values (≥ 95%–96%) identify an unknown strain to already established species name (Goris et al. 2007; Richter and Rosselló‐Móra 2009; Meier‐Kolthoff et al. 2013).

**TABLE 2:** ANI and dDDH of four *Staphylococcus* spp. with their respective type strains.

Strains	Closest type species	dDDH	ANI	Difference in GC%
*S. gallinarum* Moti Nagar	*S. gallinarum* DSM 20610^T^	93.9	99.3	0.37
*S. arlettae* Yojna Vihar 2	*S. arlettae* NCTC12413^T^	93.4	99.3	0.03
*S. haemolyticus* Yojna Vihar 1	*S. haemolyticus* ATCC 29970^T^	77.2	97.3	0.07
*S. arlettae* Naraina	*S. arlettae* NCTC12413^T^	92.2	99.1	0.04
*S. arlettae* Naraina	*S. arlettae* Yojna Vihar 2	94.9	99.4	0.07

**FIGURE 2:**
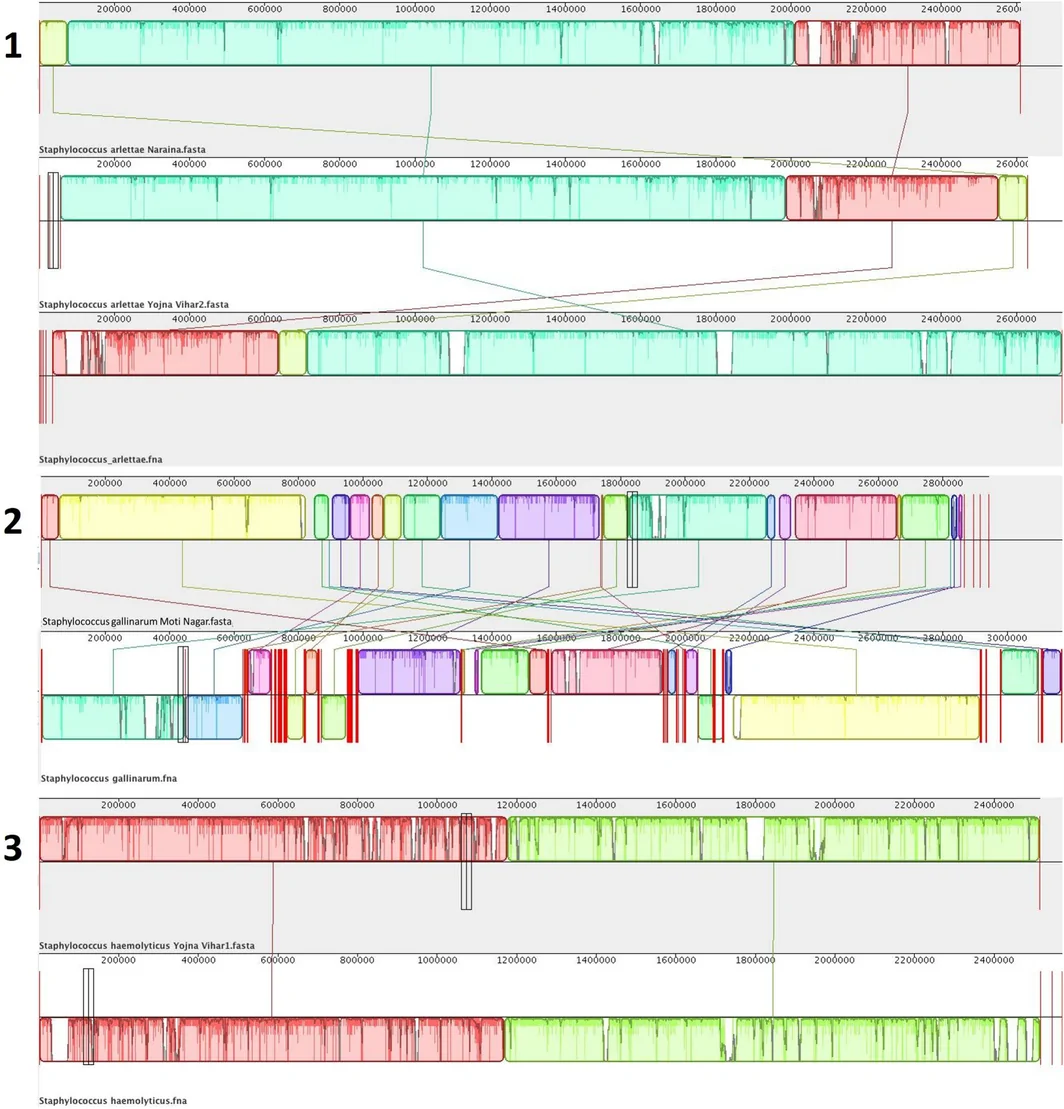
Progressive mauve alignment of the four strains along with their respective type strains: (1) alignment of *S. arlettae* NCTC12413^T^ with *S. arlettae* Naraina and *S. arlettae* Yojna Vihar 2, (2) alignment of *S. gallinarum* DSM 20610^T^ with *S. gallinarum* Moti Nagar, (3) alignment of *S. haemolyticus* ATCC 29970^T^ with *S. haemolyticus* Yojna Vihar 1.

The comparative genome analysis of the isolated strains with their respective type strain genomes was performed using Progressive Mauve to understand the genome dynamics of these air‐borne isolates (Darling et al. 2010). Local collinear blocks (LCBs) are the backbone homologous sequences present in the genomes, and discrepancies found in LCBs positions suggested rearrangements in the genomes (Darling et al. 2004). The high level of synteny observed between the genomes indicates conservation of LCBs arrangements in strains Yojna Vihar 2 and Naraina when compared with the type strain ( *S. arlettae* NCTC 12413^T^) as well as in strain Yojna Vihar 1 when compared with the type strain *S. haemolyticus* ATCC 29970^T^ (Figure 2), suggesting limited genome rearrangements. In contrast, *S. gallinarum* strain Moti Nagar showed comparatively lower synteny relative to the corresponding type strain ( *S. gallinarum* DSM 20610^T^), suggesting extensive genome rearrangements (Figure 2).

Furthermore, clusters of orthologous protein‐coding genes were analysed to understand the unique genes among the isolated strains. About 320, 30, 66, and 220 genes were uniquely present in strains *S. haemolyticus* Yojna Vihar 1, *S. arlettae* Yojna Vihar 2, *S. arlettae* Naraina, and *S. gallinarum* Moti Nagar, respectively, compared to their respective type strains of the species. This includes several transposases observed in the unique genomes of *S. haemolyticus* Yojna Vihar 1, *S. arlettae* Yojna Vihar 2, and *S. gallinarum* Moti Nagar strains; urease was detected in the unique genomes of *S. haemolyticus* Yojna Vihar 1 and *S. arlettae* Naraina; several phage proteins were observed in the unique genomes of *S. gallinarum* Moti Nagar and *S. haemolyticus* Yojna Vihar 1; permeases, hydrolases, exonucleases, and peptidases were present in the unique genomes of *S. gallinarum* Moti Nagar, *S. haemolyticus* Yojna Vihar 1, and *S. arlettae* Naraina, whereas CRISPR‐Cas machinery‐related genes were found in the *S. haemolyticus* Yojna Vihar 1 genome.

### 3.3 Movable Genetic Elements (MGEs) and Genome Plasticity

Genome plasticity contributes immensely to the evolution of the microbial world (Bohlin and Pettersson 2019). This fundamental process is also evidenced in the aid of bacterial pathogenesis by increasing host adaptability as well as the dissemination of resistance genes within the host environment (Dobrindt et al. 2010; Fajardo‐Lubián et al. 2019; Verma et al. 2019). Horizontal gene transfer via mobile genetic elements is the primary driving force in genome plasticity. Thus, we further analysed genomic islands (GIs), insertion sequences (IS), prophages in the genomes of the four airborne *Staphylococcus* spp. The Islandviewer 4 predicted 7–14 GIs in the genomes of isolated strains, exhibiting varying lengths, ranging from 4 to 65 kb and each GI containing between 2 and 56 genes. Gene annotation of GIs predicted several genes related to phage, recombinase, integrase, transposase, CRISPR‐cas, permease, hydrolase, and replication (Table S1). These findings, together with unique gene analysis, suggested that horizontal gene transfer (HGT) played a significant role in bacterial evolution and adaptability and underscored the dynamics of bacterial genomic diversity.

**FIGURE 3:**
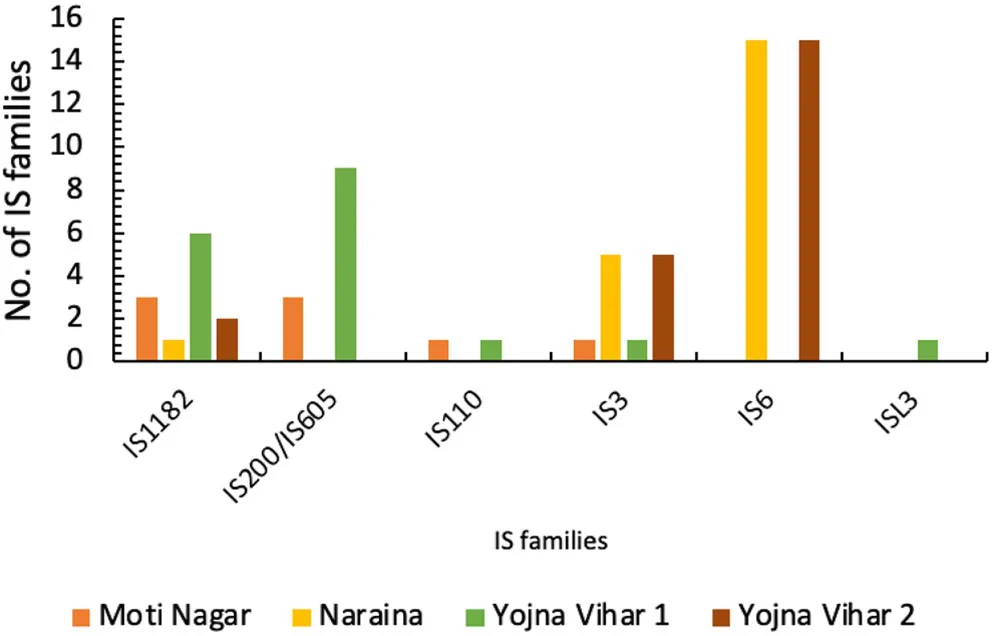
Insertion sequence families among the four *Staphylococcus* strains isolated in this study.

Further, several insertion sequences and phage sequences were detected (Figure 3, Table S2) across the strains, which further reinforced the influence of HGT in shaping genomic diversity. The analysis suggested that these air‐inhabiting *Staphylococcus* strains have undergone HGT events to persist in the polluted Delhi atmospheric ecosystem.

**FIGURE 4:**
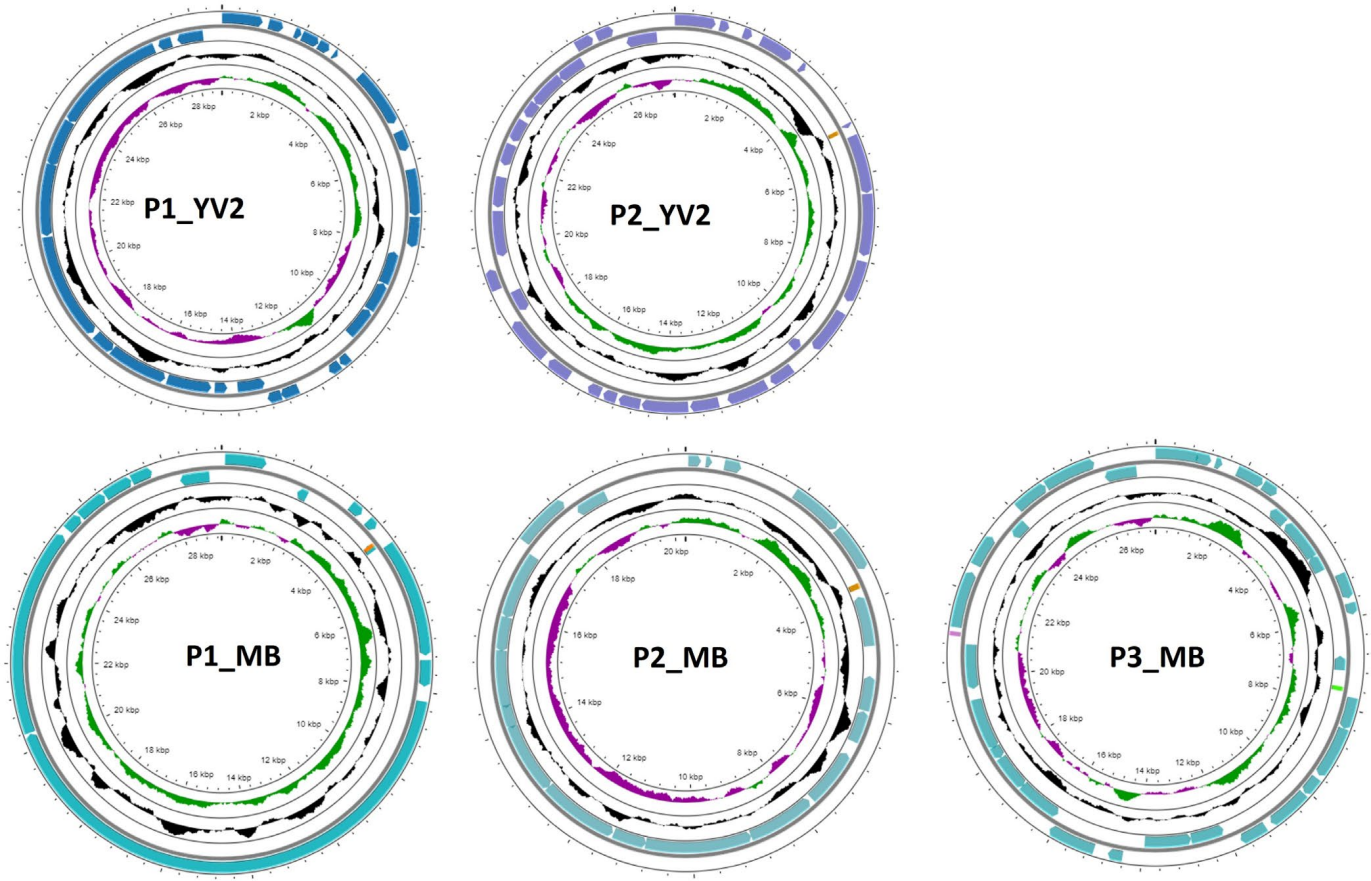
Plasmid maps of the plasmids found in *S. arlettae* Yojna Vihar 2 (P1_YV2 and P2_YV2) and *S. gallinarum* Moti Nagar (P1_MB, P2_MB, and P3_MB).

Apart from IS elements and GIs, plasmids have also played a major role in bacterial adaptability and evolution by mobilization of several accessory genes (Dionisio et al. 2019). The strain Moti Nagar contains 3 plasmids of 29.3, 27.3, and 20.2 kb with a GC content of 27.7%–29.0%. The strain Yojna Vihar 2 contains two plasmids with the lengths of 27.6 and 28.7 kb, with a GC content of 30.5% and 28.2%, respectively (Table 1, Figure 4). The plasmids P1_YV2 and P2_YV2 encode insertion sequences, transposase, hydrolase and restriction endonuclease related genes along with other replication related genes, indicating these might help the strain *S. arlettae* Yojna Vihar 2 to persist in the harsh Delhi air ecosystem, while the plasmid P1_MB of *S. gallinarum* Moti Nagar encodes a non‐ribosomal peptide synthetase (NRPS) gene cluster and P3_MB encodes a cadmium‐resistant gene.

### 3.4 Presence of the Urease Enzyme

Urease is a nickel‐dependent metalloenzyme that facilitates the hydrolysis of urea, producing ammonia (NH_3_) and carbon dioxide (CO_2_) (Krajewska 2009). For certain bacteria, urease plays a crucial role in their acid response network (Dixon et al. 1980; Konieczna et al. 2012). However, the enzyme also plays a significant role in the pathogenesis of many bacteria by helping them in niche adaptation. For example, in *Helicobacter pylori* , urease is essential for survival in the highly acidic environment of the stomach lining, where the pH can drop to 2.5. This enables the bacteria to establish and maintain chronic infection (Rutherford 2014; Schaffer et al. 2016). A few uropathogens secrete urease to survive in the lower pH conditions in the bladder (Konieczna et al. 2012; Flores‐Mireles et al. 2015; Zhou et al. 2019).

**FIGURE 5:**
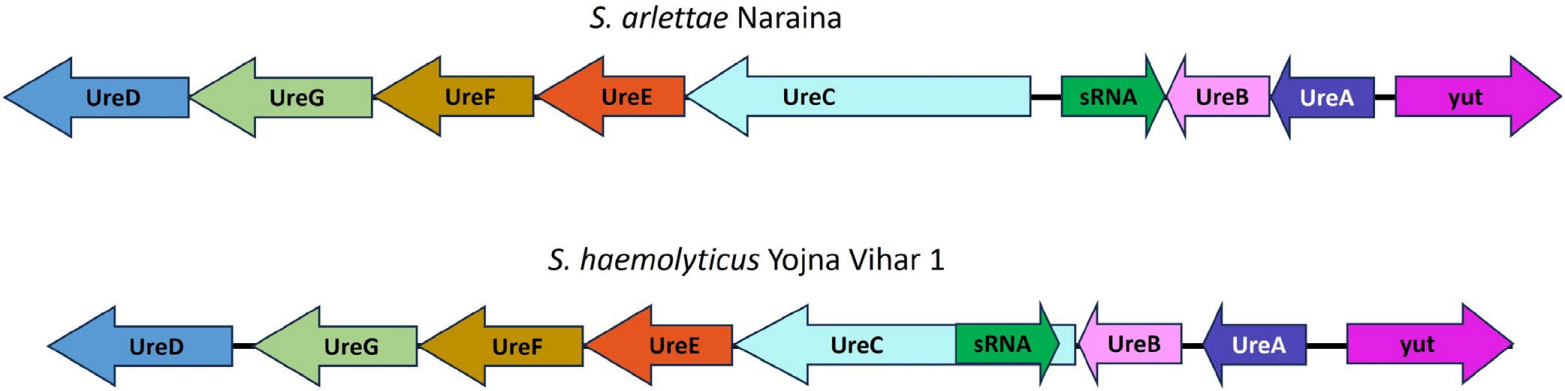
Urease gene cluster (*ureABCDEFG*) and urea transporter (*yut*) in *S. arlettae* Naraina and *S. haemolyticus* Yojna Vihar 1, isolated in this study. The images were generated using IBS v2.0 tool (https://ibs.renlab.org/#/home).

Two of our four isolates, that is, *S. haemolyticus* Yojna Vihar 1 and *S. arlettae* Naraina showed the presence of the *ureABCDEFG* gene cluster in their genomes. The KEGG analysis also supports the phenotypic results that detects these gene clusters only in strains *S. haemolyticus* Yojna Vihar 1 and *S. arlettae* Naraina (Figure 5) and are absent in the other two strains, *S. arlettae* Yojna Vihar 2 and *S. gallinarum* Moti Nagar.

Interestingly, comparative genomic analysis shows the absence of this gene cluster in their respective type strains, suggesting a possible gene gain/loss which might provide a fitness advantage to this air‐inhabitant *Staphylococcus*. However, no signatures were detected that are commonly associated with horizontal gene transfer, including adjacent mobile genetic elements, transposases, integrases, insertion sequences, atypical GC content, and genomic islands. Further, a homology search of the urease alpha subunit genes, was performed using BLASTn tool against NCBI nucleotide (nt) database. The urease alpha subunit sequence of *S. haemolyticus* Yojna Vihar 1 showed the highest similarity to three *S. haemolyticus* strains isolated from bovine mastitis, whereas *S. arlettae* Naraina showed the highest similarity to two *S. arlettae* strains isolated from human and animal hosts. Notably, the urease gene cluster was absent from most other publicly available *S. haemolyticus* and *S. arlettae* genomes. This restricted distribution may indicate the urease cluster is not universally conserved in *S. haemolyticus* or *S. arlettae* and represents lineage‐specific genomic variation. Urease is a contributory factor in the virulence of certain *Staphylococcus* species for instance, in *S. aureus* , the enzyme plays a significant role in pathogenesis by helping it persist in chronic kidney colonization in a mouse infection model, where a significant pH gradient exists and urea is an abundant nitrogen source for its persistence (Zhou et al. 2019). *S. saprophyticus* is known to cause urinary tract infection (UTI) in young females. Gatermann and group reported the contribution of *S. saprophyticus* urease to UTI pathogenesis using a rat model. Their findings suggest that urease plays a major role in UTI development by enabling their survival within the bladder tissue and contributing to inflammation (Gatermann et al. 1989). However, currently it is difficult to determine the role of the urease cluster in this air inhabitant Staphylococci. Further investigations are required to determine whether and how the urease enzyme provides any fitness advantage to these strains.

### 3.5 Prediction of Secondary Metabolites Encoding Biosynthetic Gene Clusters

**FIGURE 6:**
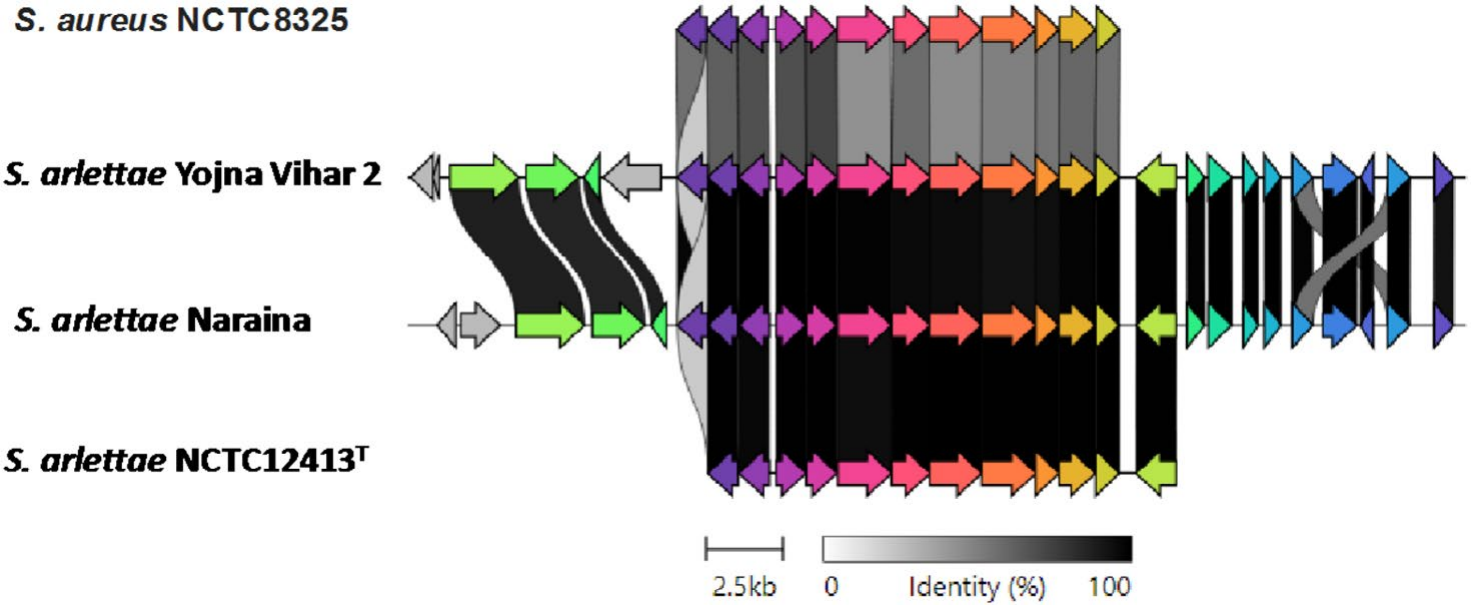
Staphyloferrin B encoding gene cluster in strain *S. arlettae* Yojna Vihar 2, *S. arlettae* Naraina, and the type strain of *S. arlettae* NCTC12413^T^.

The antiSMASH v7.0 (Blin et al. 2023) was used to identify potential biosynthetic gene clusters (BGCs) in the genomes of isolated strains. The detailed information about BGCs identified in the genomes of isolated strains is listed in Table 3. The BGCs belong to Nl‐siderophore (1–2) and cyclic‐lactone‐autoinducer (1) detected across all the strains, along with type strains of the respective species. The Nl‐siderophore‐encoded ferric‐ion‐binding compound staphyloferrin A was detected with 100% similarity in the genomes of strains *S. gallinarum* Moti Nagar and *S. haemolyticus* Yojna Vihar 1, as well as in their respective type strains. In contrast, two Nl‐siderophore clusters were identified in strains *S. arlettae* Yojna Vihar 2, *S. arlettae* Naraina, and the type strain of *S. arlettae* . Of these two clusters, one is predicted to encode staphyloferrin A with 100% identity, while the other encodes staphyloferrin B with 75% identity (Figure 6). Staphyloferrin is an iron‐chelating compound that helps bacteria obtain and assimilate iron for their growth and pathogenesis (Hammer and Skaar 2011). Further, cluster terpene, NRPS, and Type III polyketide synthase (T3PKS) were detected in both strains of *S. arlettae* and *S. gallinarum* Moti Nagar. In contrast, *S. haemolyticus* Yojna Vihar 1 lacked the NRPS cluster, while terpene and T3PKS were detected as a hybrid cluster rather than as individual clusters. A cluster of lanthipeptide was detected only in strain *S. arlettae* Yojna Vihar 2 and the type strain of *S. gallinarum* .

**TABLE 3:** List of BGCs clusters identified in four strains along with their respective type species.

BGCs cluster	*S. gallinarum* Moti Nagar	*S. gallinarum* DSM 20610^T^	*S. arlettae* Yojna Vihar 2	*S. arlettae* Naraina	*S. arlettae* NCTC12413^T^	*S. haemolyticus* Yojna Vihar 1	*S. haemolyticus* ATCC 29970^T^
Terpene	2	3	2	2	2	0	0
NI‐siderophore	1	1	2	2	2	1	1
Lanthipeptide‐class‐i	0	1	1	0	0	0	0
NRPS	1	1	1	1	1	0	0
T3PKS	1	1	1	1	1	0	0
Cyclic‐lactone‐autoinducer	1	1	1	1	1	1	1
Nucleoside	0	1	0	0	0	0	0
Terpene, T3PKS	0	0	0	0	0	1	1
RiPP‐like	0	0	0	0	0	0	1

### 3.6 Strain‐Specific Virulence Genes

The virulence and pathogenicity‐related genes were searched in the four *Staphylococcus* isolates' genomes. Our analysis uncovered distinct features in the virulence‐associated gene profiles. We found the immune evasion virulence factors, like capsular protein coding genes such as *capB* (Rausch et al. 2019), in all four *Staphylococcus* isolates (Table 4). Whereas *capC* (Rausch et al. 2019) was absent in *S. gallinarum* Moti Nagar and *galE1* was absent in *S. haemolyticus* Yojna Vihar 1 (Table 4). Enzymes like lipase (*lip*) and thermonuclease (*nuc*) (Olson et al. 2013) were also present in all four *Staphylococcus* isolates (Table 4). Serine V8 protease *sspA* was present in all except for the *S. haemolyticus* Yojna Vihar 1 (Table 4). Besides these, other virulence factor genes were differentially present in the *Staphylococcus* isolates. Although the presence of cytolysin (*cylR2*) and adherence‐related virulence factors like autolysin (*atl*), elastin‐binding protein (*ebp*), and Ser‐Asp rich fibrinogen‐binding protein (*sdrE*) genes was the most expected pathogenic strain‐specific trait found in the *S. haemolyticus* Yojna Vihar 1 (Table 4), other virulence‐related genes were also identified in other *Staphylococcus* isolates (Table 4).

**TABLE 4:** List of virulence factor genes identified in all four strains.

Vfc class	Virulence factor	Related genes	*S. gallinarum* Moti Nagar	*S. haemolyticus* Yojna Vihar 1	*S. arlettae* Naraina	*S. arlettae* Yojna Vihar 2
Adherence	Autolysin	Atl	—	1	—	—
	Elastin binding protein	Ebp	—	1	—	—
	Ser‐Asp rich fibrinogen‐binding proteins	SdrE	—	1	—	—
	LPS O‐antigen ( *P. aeruginosa* ) (*Pseudomonas*)		1	—	1	1
Enzyme	Lipase	Lip	1	1	1	1
	Serine V8 protease	SspA	1		1	1
	Thermonuclease	Nuc	1	1	1	1
Immune evasion	Capsule	Undetermined	11	6	11	11
	Polyglutamic acid capsule (*Bacillus*)	CapB	1	1	1	1
		CapC	—	2	1	1
	Polysaccharide capsule (*Bacillus*)		—	2	—	—
	Polysaccharide capsule (*Bacillus*)	GalE1	1	—	1	1
Toxin	Cytolysin (*Enterococcus*)	CylR2		1		
Serum resistance and immune evasion	LPS (*Francisella*)	WbtE	1	1	1	1
		WbtP	2	—	1	2
Serum resistance	LPS rfb locus (*Klebsiella*)		1	—	1	1
Antiphagocytosis	Capsular polysaccharide (*Vibrio*)	WcaJ	—	—	1	—
Nutritional factor	Allantoin utilization (*Klebsiella*)		4	—	4	4

### 3.7 Heterogeneity in the Presence of Antibiotic Resistance Genes

Multiple antimicrobial resistance genes (ARGs) were found in the Delhi air *Staphylococcus* isolates (Table 5). All four genomes contained genes for antibiotic efflux, such as the *Staphylococcus* drug resistance *sdrM* (provides resistance against norfloxacin, ethidium bromide, and acriflavine) (Yamada et al. 2006; Silva et al. 2023), staphylococcal efflux pump *sepA* (provides resistance against acriflavine and effluxes out ethidium bromide) (Narui et al. 2002) (Table 5). Interestingly, studies show that the *sdrM* gene is located upstream of the *sepA* gene, hinting at the possibility of the acquisition of both genes together (Narui et al. 2002). Antibiotic target alteration genes *vanT* (serine racemase; gene in the *vanG* cluster) and *vanY* (carboxypeptidase; gene in the *vanM* cluster) are known to play a role in providing resistance towards vancomycin used against *S. aureus* (Courvalin 2006; Ahmed and Baptiste 2018; Shahid et al. 2021) and were also present in all four *Staphylococcus* spp. genomes (Table 5). Although the vancomycin resistance operon/clusters (*vanG* and *vanM*) were not detected in their entirety, the presence of these genes (*vanT* and *vanY*) still suggests some degree of resistance could be observed against the drug (Rahman et al. 2025). Whereas genes like *msrA* (encodes a macrolide efflux protein), *qacG* (confers resistance against ethidium bromide and benzalkonium chloride, a common disinfectant), and *salE* (encodes a putative ABC‐F type NTPase enzyme which protects the bacterial 50S ribosomal subunit from pleuromutilin class of antibiotics) were found only in the two *S. arlettae* genomes (Heir et al. 1999; Ojo et al. 2006; Mohamad et al. 2022). Lastly, genes like *norC* (multiple‐drug resistance efflux pump, contributes to quinolone resistance) and *salD* (like *salE*, encodes a putative ABC‐F type NTPase, providing resistance against pleuromutilin class of antibiotics) were only detected in *S. gallinarum* Moti Nagar (Truong‐Bolduc et al. 2006; Mohamad et al. 2022), whereas *ARL‐2* (or *bla*_*ARL*_ gene, encodes β‐lactamase), *FosBx1* (encodes a fosfomycin inactivating enzyme), and *mphC* (encodes a phosphotransferase that inactivates macrolide class of antibiotics) were present exclusively in *S. arlettae* Yojna Vihar 2 (Matsuoka et al. 2003; Fu et al. 2016; Andreis et al. 2017), further highlighting key differences found between the two *S. arlettae* isolates.

**TABLE 5:** List of antibiotic resistance associated genes identified in four strains along with their respective gene families.

Resistance marker	Gene family	Resistance mechanism	*S. gallinarum* Moti Nagar	*S. haemolyticus* Yojna Vihar 1	*S. arlettae* Naraina	*S. arlettae* Yojna Vihar 2
*salD*	Sal‐type ABC‐F protein	Antibiotic target protection	+	−	−	−
*salE*	Sal‐type ABC‐F protein	Antibiotic target protection	−	−	+	+
*norC*	Major facilitator superfamily (MFS) antibiotic efflux pump	Antibiotic efflux	+	−	−	−
*sdrM*	Major facilitator superfamily (MFS) antibiotic efflux pump	Antibiotic efflux	+	+	+	+
*sepA*	Small multidrug resistance (SMR) antibiotic efflux pump	Antibiotic efflux	+	+	+	+
*vanT*	Glycopeptide resistance gene cluster, vanT	Antibiotic target alteration	+	+	+	+
*vanY*	VanY, glycopeptide resistance gene cluster	Antibiotic target alteration	+	+	+	+
*msrA*	Msr‐type ABC‐F protein	Antibiotic target protection	−	−	+	+
*qacG*	Small multidrug resistance (SMR) antibiotic efflux pump	Antibiotic efflux	−	−	+	+
*bla*_*ARL*_ (ARL‐2)	ARL Beta‐lactamase	Antibiotic inactivation	−	−	−	+
*FosBx1*	Fosfomycin thiol transferase	Antibiotic inactivation	−	−	−	+
*mphC*	Macrolide phosphotransferase (MPH)	Antibiotic inactivation	−	−	−	+

### 3.8 Antimicrobial Resistance Profiling of the Staphylococcus Isolates

To further confirm the resistance imparted by some of the genes found in all four *Staphylococcus* isolates, in vitro antimicrobial resistance profiling was carried out using a standard antibiotic disc assay as per the published protocols (Dureja et al. 2014). All three *Staphylococcus* isolates except *S. haemolyticus* Yojna Vihar 1, were found resistant to the β‐lactams: ampicillin, amoxiclav and methicillin. *S. haemolyticus* Yojna Vihar 1 was found to be intermediate for methicillin (Table 6, Figure S2).

**TABLE 6:** Antibiotic susceptibility testing against various classes of antibiotics.

Antibiotic class	Antibiotic	*S. arlettae* Naraina		*S. gallinarum* Moti Nagar		*S. haemolyticus* Yojna Vihar 1		*S. arlettae* Yojna Vihar 2	
		Ave diameter (mm)	SD	Ave diameter (mm)	SD	Ave diameter (mm)	SD	Ave diameter (mm)	SD
β‐lactam	Ampicillin	**16 (R)**	±2.83	**20.5 (R)**	±2.12	34 (S)	0	**18.5 (R)**	±3.53
	Amoxiclav	**13 (R)**	±4.24	**13 (R)**	±4.24	23 (S)	±1.41	**15 (R)**	±2.83
	Meropenem	26.5 (S)	±0.71	22.5 (S)	±4.94	27 (S)	±2.83	29.5 (S)	±2.12
	Ceftriaxone	23.5 (S)	±0.71	23 (S)	0	22 (S)	0	22.5 (S)	±2.22
	Cefepime	26.5 (S)	±2.12	24.5 (S)	±0.71	23.5 (S)	±0.7	29.5 (S)	±3.53
	Methicillin	**12 (R)**	±1.41	**11.5 (R)**	±0.71	**16.5 (I)**	±0.71	**11.5 (R)**	±2.21
Aminoglycosides	Kanamycin	21 (S)	±4.24	17.5 (S)	±2.12	20.5 (S)	±2.12	23.5 (S)	±2.42
	Gentamicin	22 (S)	±1.43	19 (S)	±2.83	23 (S)	±2.82	24.5 (S)	±0.71
Sulfonamides	Sulphafurazole	27 (S)	0	26 (S)	±1.42	21.5 (S)	±0.71	29.5 (S)	±3.56
	Co‐trimoxazole	24.5 (S)	±2.13	27.5 (S)	±2.1	19 (S)	±1.45	25.5 (S)	±0.7
Fluoroquinolone	Ciprofloxacin	25 (S)	±2.82	22 (S)	0	21.5 (S)	±3.55	25 (S)	±2.82
Glycopeptide	Vancomycin	**15.5 (R)**	±0.7	**13.5 (R)**	±0.7	**13.5 (R)**	±0.71	**14 (R)**	±1.41
Tetracycline	Tetracycline	29 (S)	±1.42	28 (S)	±1.41	25 (S)	±2.83	30 (S)	±2.82
Chloramphenicol	Chloramphenicol	25 (S)	±1.41	23.5 (S)	±0.71	24.5 (S)	±0.71	27 (S)	±2.85

*Note:* The values are in bold to highlight the strains showing resistance to antibiotics.

Abbreviations: I, intermediate; R, resistant; S, susceptible.

The broth microdilution was further employed for determining the MIC of vancomycin against *Staphylococcus* spp., as it is still considered the last line of treatment against *Staphylococcus* infections (Othman et al. 2018). Our results showed that the MIC of vancomycin against *S. haemolyticus* (Yojna Vihar 1), *S. arlettae* (Yojna Vihar 2), *S. arlettae* (Naraina), and *S. gallinarum* (Moti Nagar) was found at 8 μg/mL. 2 μg/mL, 2 μg/mL and 8 μg/mL, respectively (Table 7). As per the MIC breakpoint, mentioned in CLSI‐M100, two strains of *Staphylococcus* spp. ( *S. arlettae* Yojna Vihar 2 and *S. arlettae* Naraina) were found sensitive to vancomycin, as their MICs are within the sensitive range ≤ 4 μg/mL and the other two strains were found intermediate, as their MICs are within the range of 8–16 μg/mL.

**TABLE 7:** MIC of vancomycin against *Staphylococcus* spp. isolates and their susceptibility classification according to CLSI M100 (2024) breakpoints.

Name of the bacterial strains	MIC (μg/mL)
*S. haemolyticus* Yojna Vihar 1	8
*S. arlettae* Yojna Vihar 2	2
*S. arlettae* Naraina	2
*S. gallinarum* Moti Nagar	8

### 3.9 Phenotypic Microarray of the Staphylococcus Isolates

As a high‐throughput technology, phenotypic microarray has been used not only to study the cellular metabolic phenotypes among various strains of the same species, but it is also considered a reliable method for species identification and classification (Klingler et al. 1992; von Eiff et al. 2006). Here we utilized the phenotypic microarray analysis to observe growth differences in specific carbon source usages by the four *Staphylococcus* air isolates. While all four strains showed growth in common sugars like D‐glucose, D‐mannose, D‐fructose, D‐galactose, dextrin, D‐maltose, sucrose, and so forth (Table 8), many differences were observed among them as well. For example, *S. gallinarum* Moti Nagar failed to grow in fucose, α‐D‐Lactose, N‐Acetyl Neuraminic acid, N‐Acetyl‐D‐galactosamine, L‐rhamnose, and D‐malic acid. While both *S. arlettae* strains showed similar growth patterns in most of the substrates, *S. arlettae* Naraina showed compromised growth compared to its Yojna Vihar 2 counterpart in the presence of D‐malic acid and methyl pyruvate (Table 8). The isolated strain of the known pathogenic species, *S. haemolyticus* Yojna Vihar 1 showed no growth in N‐acetyl‐D‐galactosamine, D‐malic acid, D‐galacturonic acid, and L‐alanine, whereas it was also the only strain to grow in L‐rhamnose. Among the four, the most competent strain that grew on the maximum substrates was *S. arlettae* Yojna Vihar 2 (Table 8).

**TABLE 8:** Phenotypic microarray of *Staphylococcus* air isolates.

Carbon source	*S. gallinarum* Moti Nagar	*S. haemolyticus* Yojna Vihar 1	*S. arlettae* Naraina	*S. arlettae* Yojna Vihar 2
D‐Glucose	+	+	+	+
3‐Methyl‐glucose	+	+	+	+
D‐Fucose	−	+	+	+
L‐Fucose	−	+	+	+
D‐Mannose	+	+	+	+
D‐Fructose	+	+	+	+
D‐Galactose	+	+	+	+
Dextrin	+	+	+	+
D‐Maltose	+	+	+	+
D‐Trehalose	+	+	+	+
D‐Cellobiose	+	+	+	+
Gentiobiose	+	+	+	+
Sucrose	+	+	+	+
D‐Turanose	+	+	+	+
Stachyose	+	+	+	+
D‐Raffinose	+	+	+	+
α‐D‐Lactose	−	+	+	+
D‐Melibiose	+	+	+	+
β‐Methyl‐D‐glucoside	+	+	+	+
D‐Salicin	+	+	+	+
N‐Acetyl‐D‐glucosamine	+	+	+	+
N‐Acetyl‐β‐D‐mannosamine	+	+	+	+
N‐Acetyl‐D‐galactosamine	−	−	+	+
N‐Acetyl Neuraminic acid	−	+	+	+
1% Sodium lactate	+	+	+	+
L‐Lactic acid	+	+	+	+
Inosine	+	+	+	+
D‐Sorbitol	+	+	+	+
Gelatin	+	+	+	+
D‐Arabitol	+	+	+	+
D‐Cellobiose	+	+	+	+
D‐Trehalose	+	+	+	+
D‐Turanose	+	+	+	+
Methyl pyruvate	+	+	−	+
D‐Malic acid	−	−	−	+
D‐Glucose‐6‐phosphate	+	+	+	+
D‐Fructose‐6‐phosphate	+	+	+	+
L‐Rhamnose	−	+	−	−
D‐Galacturonic acid	+	−	+	+
D‐Gluconic acid	+	+	+	+
L‐Malic acid	+	+	+	+
Glycerol	+	+	+	+
D‐Saccharic acid	+	+	+	+
β‐methyl‐D‐glucoside	+	+	+	+
Amino Acids				
L‐Alanine	+	−	+	+
L‐Aspartic acid	+	+	+	+
L‐Glutamic acid	+	+	+	+
L‐Histidine	+	−	+	+
L‐Serine	+	+	+	+
L‐Arginine	+	+	+	+
L‐Pyroglutamic acid	+	+	+	+
Glycyl‐L‐Proline	+	+	+	+
D‐Serine	+	+	−	−
D‐Aspartic acid	−	−	+	+
pH				
pH 5.0	+	+	−	−
pH 6.0	+	+	+	+
Salt				
NaCl 1%	+	+	+	+
NaCl 4%	+	+	+	+
NaCl 8%	+	+	+	+
Lithium chloride	+	+	+	+
Sodium butyrate	+	+	+	+
Sodium bromate	+	−	+	+

### 3.10 Black Carbon Increases the Exopolysaccharide Production in the Staphylococcus Isolates

Biofilms are known to provide a safe environment for the embedded bacterial cells. To investigate the ability of the *Staphylococcus* spp. isolated from the Delhi air to survive in the environmental conditions, and hence to study their adaptability for inhabiting the resident human population, the impact of black carbon on biofilm formation by the *Staphylococcus* spp. was observed as previously described (Hussey et al. 2017). All four *Staphylococci* strains were exposed to a fixed concentration of 100 μg/mL of BC during the biofilm formation. As the exact concentration of the BC pollutant in the Delhi population is not yet known, we selected the previously selected concentration by Hussey and group (Hussey et al. 2017).

**FIGURE 7:**
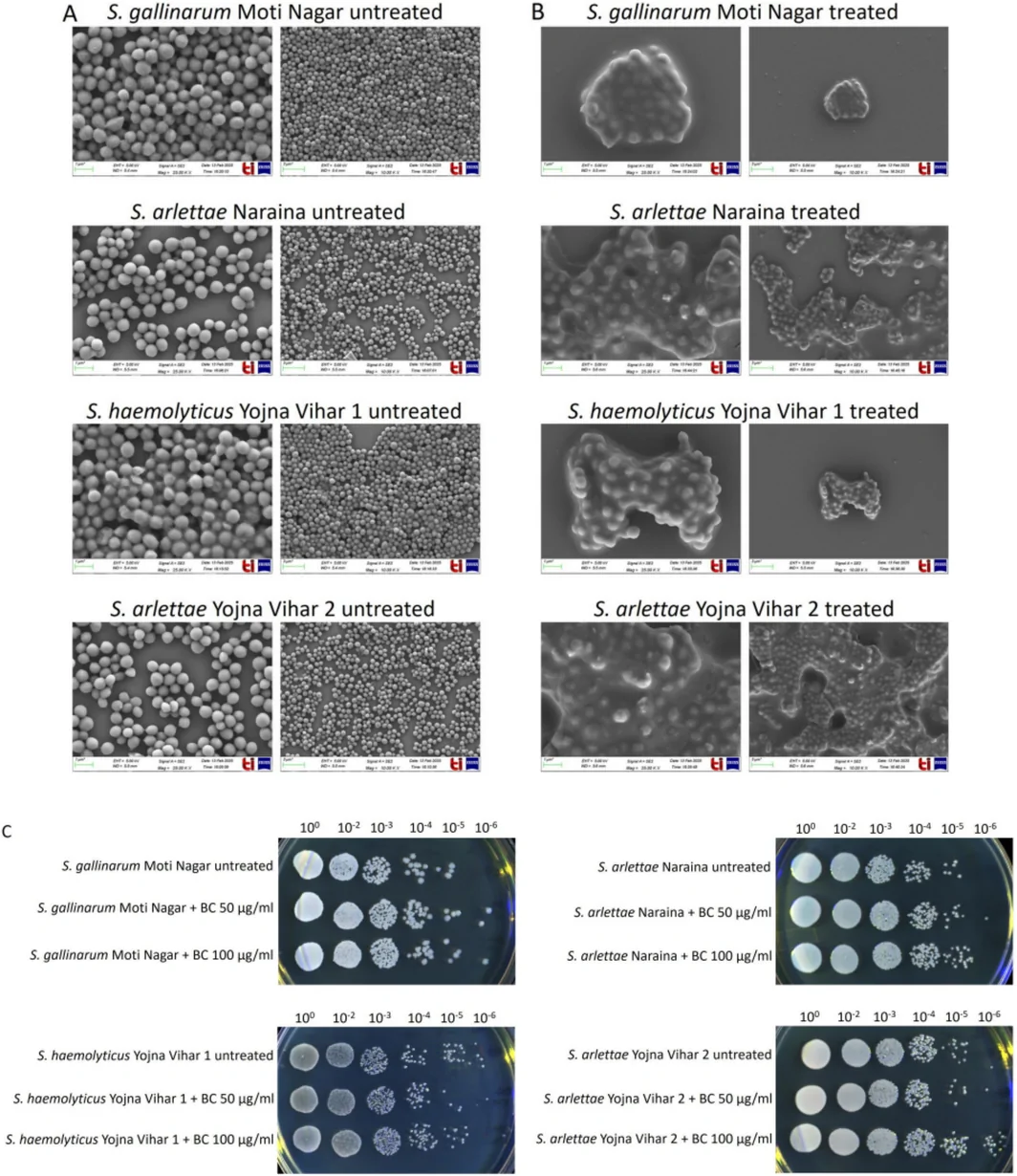
Biofilm formation by *Staphylococcus* isolates in the presence of black carbon. (A) Scanning electron microscope (SEM) images of all four *Staphylococcus* strains showing biofilm formation in BHI. (B) SEM images of all strains showing biofilm formation in the presence of black carbon (100 μg/mL) particles. (C) Serially diluted cells removed from the biofilm were spotted on BHI agar plates.

In the scanning electron microscopy images, BC was observed to induce a drastic change in the biofilm architecture of all four Delhi isolates of *Staphylococcus* spp. Biofilms formed in the presence of BC were found to be highly engulfed in the secreted EPS, with lots of protrusions forming in the cell lawn, whereas biofilms formed in the absence of BC were observed to produce very little EPS, with individual cells being visible under the microscope (Figure 7A,B). Interestingly, no change was observed in the number of cells in these conditions (Figure 7C).

**FIGURE 8:**
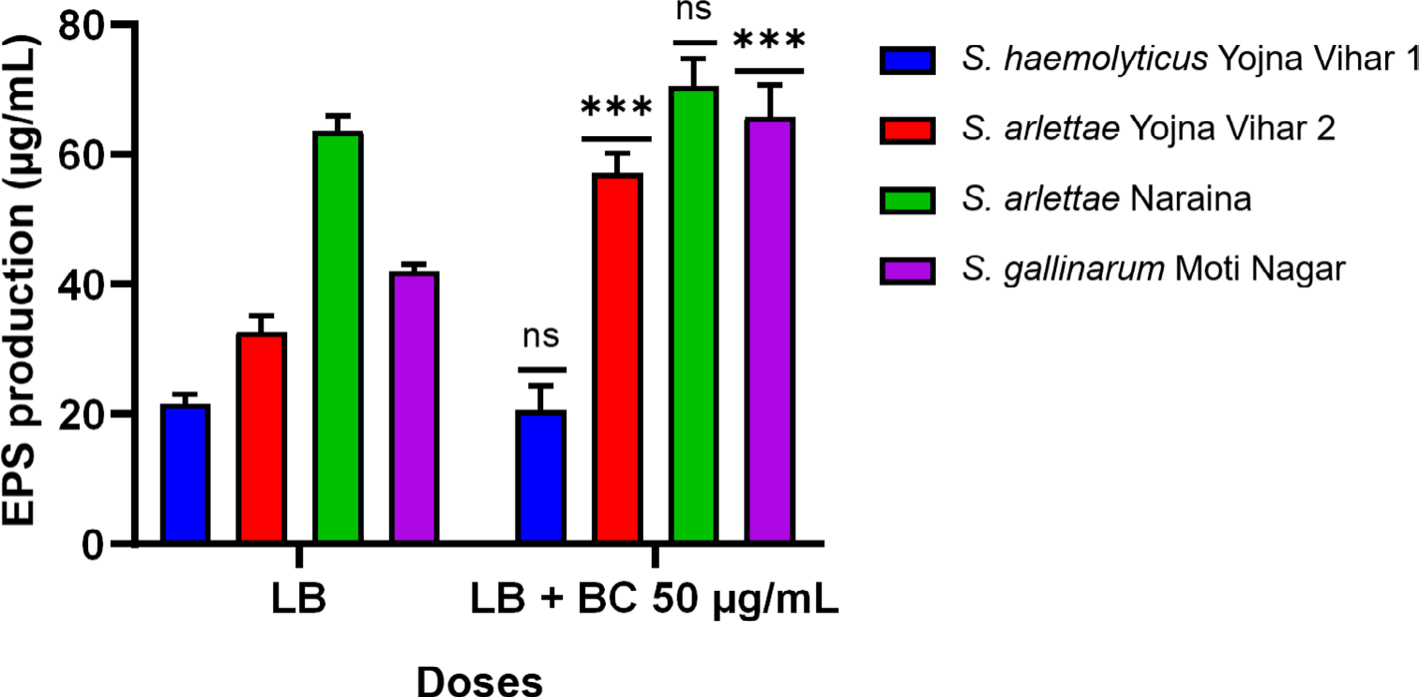
Quantification of EPS from the biofilm of *Staphylococcus* spp. The production of EPS in the presence and absence of black carbon 50 μg/mL was quantified. Significance levels of BC 50 μg/mL treated bacterial samples with respect to their counterparts grown in LB were determined by three‐way ANOVA with Sidak's multiple comparisons test: *p* ≥ 0.05 = ns, *p* < 0.001 = ***.

To further quantify the extracellular polymeric substances (EPS) that form the structural backbone of staphylococcal biofilms, EPS concentrations were measured in all four isolates under baseline (LB broth) and black carbon (BC)‐supplemented (50 μg/mL) conditions following established protocols (Goswami et al. 2025; Paul et al. 2026). All isolates were confirmed EPS producers (Figure 8). Baseline EPS concentrations were 21.67, 32.76, 63.54, and 42.08 μg/mL for *S. haemolyticus* (Yojna Vihar 1), *S. arlettae* (Yojna Vihar 2), *S. arlettae* (Naraina), and *S. gallinarum* (Moti Nagar), respectively. Upon BC exposure, EPS production increased markedly in three isolates— *S. arlettae* (Yojna Vihar 2; 57.08 μg/mL), *S. arlettae* (Naraina; 70.47 μg/mL), and *S. gallinarum* (Moti Nagar; 65.78 μg/mL), while *S. haemolyticus* showed a marginal decrease (20.63 μg/mL), suggesting a species‐specific regulatory response. Black carbon has previously been shown to drastically alter bacterial biofilm development, inducing structural and compositional changes that enhance tolerance to antibiotics and proteolytic degradation, corroborating the BC‐driven EPS upregulation observed here in the majority of isolates.

## 4 Discussion

Air is one of the five classical elements, and clean air is vital for human health, ecosystems, and the environment overall. Air pollution remains a major global problem, causing millions of deaths each year. According to the State of Global Air 2024 report, nearly 8.1 million deaths occurred worldwide in 2021 due to air pollution (https://www.stateofglobalair.org/resources/report/state‐global‐air‐report‐2024). Alarmingly, the air quality in New Delhi, the capital of India, ranks among the poorest in the world (Chen 2025). While numerous investigations have been conducted on Delhi's air quality, they primarily focused on analysing its chemical composition. On the other hand, very few have studied its microbial composition, including a recent study by Mukhopadhyay and co‐workers (Kumari et al. 2025). The results presented by Kumari et al. 2025 further corroborated our findings of the *Staphylococcus* spp. being present in the outdoor air of the megacity. In this study, we identified several bacterial species from Delhi air samples, and eventually, we kept our focus on *Staphylococcus* species because of their pathogenic potential.

*Staphylococcus arlettae* (SAR) was isolated for the first time from the skin of farm animals (Schleifer et al. 1984). Though considered a commensal organism, recently SAR and other coagulase‐negative *Staphylococcus* (CoNS) species (e.g., *S. haemolyticus* ) are reported to be increasingly associated with human and animal infections (Lavecchia et al. 2019; Eltwisy et al. 2020; Bernier Gosselin et al. 2019). Alarmingly, a SAR strain SA‐01 isolated from poultry was documented to harbour a plasmid encoding nine antibiotic resistance genes (Liu et al. 2017). Likewise, *S. haemolyticus* , another CoNS of the commensal category, also emerges as an increasing threat in the case of immunocompromised patients, particularly those with medical implants (Czekaj et al. 2015). The organism deploys multiple virulence factors to cause severe skin infections and contribute to the progression of diabetic foot ulcers (Basso et al. 2014; Skov et al. 2000).

Multiple studies have documented the airborne transmission of drug‐resistant *Staphylococcus* species, particularly *S. aureus* , and their significant public health risk (Madsen et al. 2018; Kozajda et al. 2019; Singh et al. 2021; Kumari et al. 2025). A recent study has illustrated the prevalence and resistance profile of airborne *Staphylococci* in various urban settings in Delhi. Of the eight staphylococcal species, *Staphylococcus epidermidis* and *S. arlettae* were the most abundant. Further analysis of antibiotic resistance genes (ARG) revealed that 73% of methicillin‐resistant staphylococci isolates exhibited multidrug resistance, showing resistance to macrolides, β‐lactams, and other commonly used antibiotics (Kumari et al. 2025). Similarly, in our study, we observed resistance towards β‐lactams (ampicillin, amoxiclav, and methicillin) and glycopeptides (vancomycin). The burden of vancomycin‐resistant *Staphylococcus* species ( *S. aureus* and coagulase‐negative staphylococci) is a significant global public health concern due to limited treatment options, high healthcare costs, and increased patient morbidity and mortality (Cong et al. 2019; Shariati et al. 2020; Selim et al. 2022). Vancomycin resistance not only poses a public health concern but also an emerging threat to animal health (Aqib and Alsayeqh 2022).

Alarmingly, particulate matter (PM2.5) plays a critical role in the dissemination of SARS‐CoV‐2 (Nor et al. 2021). Air pollution and antimicrobial resistance are also recognized as two emerging challenges threatening global health (Xiu and Hu 2025). Intriguingly, a direct correlation has been demonstrated between the level of dust in various indoor and outdoor environments and the concentration of airborne methicillin resistant *Staphylococcus aureus* (MRSA) (Madsen et al. 2018; White et al. 2020). Our current work on isolating drug‐resistant *Staphylococcus* species from Delhi air samples further supports the ongoing threat posed by air as a carrier of antimicrobial resistance. The findings of our current study also highlight the antibiotic resistance towards β‐lactams like ampicillin, amoxycillin, and methicillin, and partial resistance towards vancomycin observed in 50% of the *Staphylococcus* isolates found in Delhi air. The MIC of vancomycin was determined for all four *Staphylococcus* spp. isolates by broth microdilution and interpreted according to CLSI M100 (2024) breakpoints (Table 7). *S. haemolyticus* Yojna Vihar 1 and *S. gallinarum* Moti Nagar exhibited MIC values of 8 μg/mL each, placing them in the intermediate susceptibility category (MIC 8–16 μg/mL). In contrast, *S. arlettae* Yojna Vihar 2 and *S. arlettae* Naraina demonstrated MIC values of 2 μg/mL each, classifying both isolates as susceptible (MIC ≤ 4 μg/mL). The intermediate resistance observed towards vancomycin, combined with the presence of genes (*vanT* and *vanY*) is particularly interesting, as the complete vancomycin resistance operon was not found in the genomes. As evidenced by the case of *Staphylococcus aureus* , intermediate vancomycin resistance can arise from progressive mutations in cell wall regulatory genes. These genetic changes trigger thickening of the cell wall and the production of “false” binding targets, effectively trapping the antibiotic on the outer surface and preventing it from reaching its intracellular target (McGuinness et al. 2017). Therefore, the intermediate susceptibility of *S. haemolyticus* Yojna Vihar 1 and *S. gallinarum* Moti Nagar against vancomycin could be due to an altered cell wall. Similarly, no *mecA* or related methicillin‐resistance genes were detected in the genomes, despite the strains exhibiting a methicillin‐resistant phenotype. This discrepancy may indicate *mec*‐independent resistance mechanisms, including alterations in PBP4 or other penicillin‐binding proteins that reduce bacterial affinity for β‐lactam antibiotics (Banerjee et al. 2010; Elhassan et al. 2015; Cikman et al. 2019). This requires further investigation.

Vancomycin is usually considered the last line of defence against *Staphylococcus* infections. Our results show that the current situation could worsen over time (Ahmed and Baptiste 2018; Rahman et al. 2025). Further studies are required to understand the resistance profile and virulence potential of such air isolates.

Another critical issue is the formation of aerial biofilm. Biofilms are widely recognized as “hotspots” for HGT, serving as a platform for the efficient exchange of genetic material among microorganisms. This exchange is a major driver of bacterial evolution and adaptation, including the rapid spread of traits like antibiotic resistance and virulence factors (Nkemngong and Teska 2024). Moreover, aerial biofilm is also responsible for long‐distance transportation of the microbes (Lang‐Yona et al. 2025). We evidenced strong biofilm development by our drug‐resistant *Staphylococcus* species in the presence of black carbon, further underpinning the issues related to the transfer of genetic material within biofilm and long‐distance transportation of these isolates through particulate matter. This warrants further investigation.

## Author Contributions

**Himanshu Sen:** writing – original draft, methodology, data curation, conceptualization, investigation, validation, visualization, writing – review and editing, formal analysis. **Joyasree Das:** investigation, writing – original draft, validation, visualization, writing – review and editing, methodology, software, data curation. **Suman Paul:** validation, visualization, data curation. **Manpreet Kaur:** validation, formal analysis, visualization. **Amareshwar Vodapalli:** investigation, validation, formal analysis. **Surajit Bhattacharjee:** formal analysis, resources, writing – review and editing. **Manoj Raje:** conceptualization, project administration, resources, writing – review and editing. **Srinivasan Krishnamurthi:** formal analysis, supervision, resources, visualization, funding acquisition. **Saumya Raychaudhuri:** conceptualization, funding acquisition, writing – review and editing, supervision, resources, project administration, formal analysis.

## Funding

This work was partly supported by grants from the CSIR‐IMTECH in‐house OLP 151, OLP 193 to SR and CSIR FBR project FBR050301 to SRC and SKM and a DST grant to MR who was also partially supported by an Emeritus Scientist fellowship from the Indian Council of Medical Research.

## Conflicts of Interest

The authors declare no conflicts of interest.

## Supporting information


**Figure S1:** 16S rRNA based phylogenetic tree. The evolutionary history was inferred using the Neighbour‐Joining method. The optimal tree with the sum of branch lengths = 0.31389318 is shown. The percentage of replicate trees in which the associated taxa clustered together in the bootstrap test (1000 replicates) is shown next to the branches. The tree is drawn to scale, with branch lengths in the same units as those of the evolutionary distances used to infer the phylogenetic tree. The evolutionary distances were computed using the Maximum Composite Likelihood method and are in the units of the number of base substitutions per site. The analysis involved 93 nucleotide sequences. All positions containing gaps and missing data were eliminated. There was a total of 857 positions in the final dataset. Evolutionary analyses were conducted in MEGA7. The filled circles (●) represent the nodes recovered in the neighbour‐joining, and maximum‐likelihood algorithms, while the filled triangles (▲) represent the nodes that differ between the neighbour‐joining and maximum‐likelihood algorithms. Strains highlighted in blue represent those isolated in this study.

**Figure S2:** Kirby‐Bauer disk diffusion susceptibility test for methicillin and vancomycin. The Kirby‐Bauer antibiotic disk assay was performed with the four Delhi air *Staphylococcus* isolates along with *S. aureus* ATCC 25923. The antibiotic discs (HiMedia) taken, were methicillin (5) and vancomycin (30). The plate images shown here are best representatives of the experiment performed in biological triplicates.

**Table S1:** List of genomic Islands detected in the genomes of *S. arlettae* Naraina, *S. arlettae* Yojna Vihar 2, *S. haemolyticus* Yojna Vihar 1 and *S. gallinarum* Moti Nagar.

**Table S2:** Putative prophage regions identified in the genomes of *S. arlettae* Naraina, *S. arlettae* Yojna Vihar 2, *S. haemolyticus* Yojna Vihar 1, and *S. gallinarum* Moti Nagar.

## Data Availability

The GenBank accession numbers of the sequenced genomes are as follows: *S. gallinarum* Moti Nagar (CP182780), *S. arlettae* Yojna Vihar 2 (CP182778), *S. haemolyticus* Yojna Vihar 1 (CP182779), and *S. arlettae* Naraina (CP182784) genomes respectively. The accession numbers of plasmid DNA sequences are as follows: *S. arlettae* strain Yojna Vihar 2 [P1_YV2 (CP182776), P2_YV2 (CP182777)] and *S. gallinarum* Moti Nagar [namely P1_MB (CP182781), P2_MB (CP182782), P3_MB (CP182783)]. The microbiological assays data are available at request with the corresponding author: saumya@rocketmail.com; s.raychaudhuri@csir.res.in.
